# Transcriptome Analysis in *Haematococcus pluvialis*: Astaxanthin Induction by Salicylic Acid (SA) and Jasmonic Acid (JA)

**DOI:** 10.1371/journal.pone.0140609

**Published:** 2015-10-20

**Authors:** Zhengquan Gao, Yan Li, Guanxun Wu, Guoqiang Li, Haifeng Sun, Suzhen Deng, Yicheng Shen, Guoqiang Chen, Ruihao Zhang, Chunxiao Meng, Xiaowen Zhang

**Affiliations:** 1 School of Life Sciences, Shandong University of Technology, Zibo, 255049, P.R. China; 2 Yellow Sea Fishery Research Institute, Chinese Academy of Fishery Sciences, Qingdao, 266071, P.R. China; 3 College of Marine and Environmental Sciences, James Cook University, Douglas, 4811, Australia; 4 School of Food and Agriculture Sciences, University of Queensland, St. Lucia, 4072, Australia; Institute of Crop Sciences, CHINA

## Abstract

*Haematococcus pluvialis* is an astaxanthin-rich microalga that can increase its astaxanthin production by salicylic acid (SA) or jasmonic acid (JA) induction. The genetic transcriptome details of astaxanthin biosynthesis were analyzed by exposing the algal cells to 25 mg/L of SA and JA for 1, 6 and 24 hours, plus to the control (no stress). Based on the RNA-seq analysis, 56,077 unigenes (51.7%) were identified with functions in response to the hormone stress. The top five identified subcategories were cell, cellular process, intracellular, catalytic activity and cytoplasm, which possessed 5600 (~9.99%), 5302 (~9.45%), 5242 (~9.35%), 4407 (~7.86%) and 4195 (~7.48%) unigenes, respectively. Furthermore, 59 unigenes were identified and assigned to 26 putative transcription factors (TFs), including 12 plant-specific TFs. They were likely associated with astaxanthin biosynthesis in *Haematococcus* upon SA and JA stress. In comparison, the up-regulation of differential expressed genes occurred much earlier, with higher transcript levels in the JA treatment (about 6 h later) than in the SA treatment (beyond 24 h). These results provide valuable information for directing metabolic engineering efforts to improve astaxanthin biosynthesis in *H*. *pluvialis*.

## Introduction

*Haematococcus pluvialis* is a unicellular freshwater microalga and has been well known as a rich, natural resource of the high-value carotenoid astaxanthin. Its astaxanthin (3, 3'-dihydroxy-ß-carotene-4, 4'-dione) is the most powerful natural antioxidant and is highly demanded in the nutraceutical and aquaculture fields [1]. Although it can also be produced by other resources (e.g., yeasts, higher plants and aquaculture wastes), the astaxanthin production of *Haematococcus* is up to 4% of cellular dry weight (DW), which is much higher and more efficient than other sources. Therefore, the astaxanthin of *H*. *pluvialis* is of huge commercial importance and has attracted significant scientific attention in recent years [2].

Since the astaxanthin can be dramatically accumulated in the algal cells when *H*. *pluvialis* is exposed to stress, such as high light intensity, high temperature, low levels of oxygen and/or chemical substances [3–7], astaxanthin accumulation is also part of a self-protection process [2]. However, the genetic details and regulations of the induced biosynthetic routes leading to the astaxanthin accumulation are still not well understood. Therefore, a genome-wide survey, along with differential gene expression analysis, would be necessary to identify all genes involved in *H*. *pluvialis* astaxanthin biosynthesis. Particularly, comparative analysis of genes’ expression profiles can facilitate identifying key regulatory genes, and also associated metabolic pathways. Regarding this, comparative enzymatic inductions of Jasmonic acid (JA) and salicylic acid (SA) can provide a useful approach to investigate the expression profile details.

JA and SA are important hormones in stress-related signaling pathways of plants [8], especially in plants’ defense via phytohormone signal transduction [9]. Similarly, hormones SA and JA can also induce self-protection on microalga *H*. *pluvialis*. Our previous studies have reported that they both can induce higher transcriptional expression levels of carotenogenesis genes (*ipi-1*, *ipi-2*, *psy*, *pds*, *lyc*, *bkt*2, *crt*R-B and *crt*O), resulting in higher astaxanthin productivity on *H*. *pluvialis* [6, 7]. However, the relevant genes showed different genetic expression patterns when *H*. *pluvialis* was stressed by the same dose of JA and SA. For example, with JA induction, *psy*, *pds*, *crt*R-B, *lyc*, *bkt2* and *crt*O were up-regulated at the transcriptional level only, and *ipi-1*, *ipi-2* were up-regulated at both transcriptional and post-transcriptional levels. Nevertheless, with SA stress, genes of *ipi-1*, *ipi-2*, *psy*, *crt*R-B, *bkt* and *crt*O were up-regulated at transcriptional level, *lyc* was at post-transcriptional level, and only *pds* was at both transcriptional and post-transcriptional levels. Moreover, the induced astaxanthin production was also different between SA and JA inductions [6, 7]. To date, how microalgae respond to hormone stress at the molecular level is largely limited and the relevant pathways have also not been fully documented. Therefore, it is still unclear whether there are any different genomic and metabolic features of astaxanthin biosynthesis between SA and JA enzymatic inductions on *H*. *pluvialis*.

To obtain microalgae functional genomics information, transcriptome sequencing represents an essential and efficient approach [10]. In fact, microalgal transcriptome research has made progress in recent years. For example, the sequencing and *de novo* transcriptome assembly on *Dunaliella* microalga have identified the key pathways and important genes for biofuel production [10]. The transcriptome analysis has been conducted on a cold Arctic strain of *Chlamydomonas* sp. [11], showing the responsible genes for cold tolerance, photosynthesis activity and fatty acid (FA) biosynthesis, and also identifying the putative homologs of antifreeze protein in microalgae. Similarly, the lipid metabolism on *Nannochloropsis sp*. [12], H_2_ photoproduction in *Chlamydomonas moewusii* and *C*. *reinhardtii* during the dark anaerobic induction [13] have also been investigated via their individual transcriptomic profiles for better understanding on the functional genes and associated pathways. Coupled with an advanced next-generation sequencing method, these transcriptome sequencings only target coding DNA. With less sequencing requirements, transcriptome sequencing can have large transcriptome coverage depth and largely facilitate the *de novo* assembly of transcriptomes [10].

RNA sequencing (RNA-seq) is a popular deep sequencing technology for analyzing entire transcriptomes. High-through DNA sequencing can produce a library of short cDNA reads that can then be aligned to a reference genome or transcriptome or can be assembled *de novo* without knowing the genome sequence of a given organism [2]. Therefore, RNA-seq is very attractive for the study on *H*. *pluvialis*. RNA-seq cannot only provide a genome-scale transcription map of transcriptome structure, but also the level of transcriptional expression of individual genes [2, 14]. These techniques can significantly improve our understanding of astaxnathin biosynthesis in *H*. *pluvialis* at the genetic modulation level.

In the present study, the transcriptomes of *H*. *pluvialis* were sequenced using Illumina Hiseq2000, to inspect the regulation of metabolic pathways and differential expressions of genes under JA or SA stress. RNA-Seq was applied for transcriptomic profiling. The assembled sequences and singletons were subjected to BLAST similarity searches and annotated with Gene Ontology (GO), Encyclopedia of Genes and Genomes (KEGG) and Kyoto Orthology Genomes (KOG) identifiers. Based on the NCBI database, the results of Blastp and GoPipe were used to interpret the functions of the assembled unigenes. A combination of Transcriptome Factors (TFs) would demonstrate the hormonal signal pathways regulated by SA and JA inductions. The comparative analyses of JA and SA inductions at transcriptional levels will provide additional valuable information for deciphering the transcriptional regulatory networks in *H*. *pluvialis*, and directing metabolic engineering efforts towards valuable astaxanthin production.

## Results and Discussion

### Sequencing and assembling analyses in *H*. *pluvialis*

**Fig 1:**
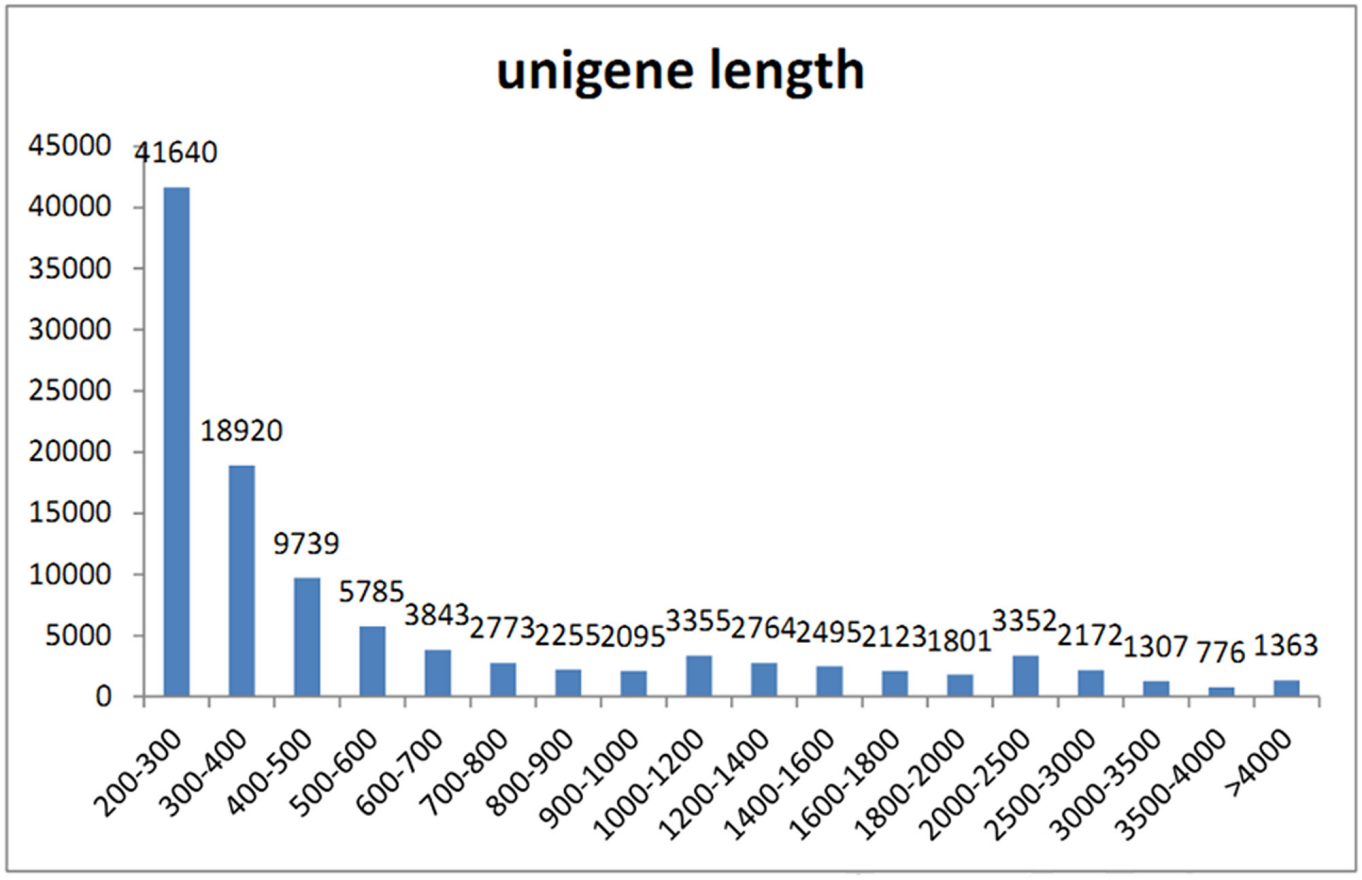
The distribution of unigene length on *H*.*pluvialis*. The -ais indicates gene length and the y-ais indicates gene number.

The sequencing yielded about 12,000,000 raw reads in each sample (Table 1) and the accession number of the relevant sequencing data have been reported to GSE71986. The pre-assembly process of trimming and cleaning for quality control produced about 95% clean reads with 100 nucleotides of average length in each. These clean reads were further assembled into contiguous sequences (contigs), and the longest sequence of a subgroup was used as the unigene reference. As a result, a total of 108,558 unigenes was obtained, with lengths ranging from 201 bp to 16,073 bp, but the majority was between 200 bp and 500 bp in all the samples (Fig 1). A similar study on *H*. *pluvialis* obtained 1,453,995 contigs [2], which was many more than the value in this study. Since they used photooxidative stress for astaxanthin induction [2], the different number of contigs identified in our study further indicates that the metabolic modulation induced by hormone stress should be also different.

**Table 1:** Summary of Illumina sequencing results in *H*. *pluvialis*.

Sample	N	JA1	JA6	JA24	SA1	SA6	SA24
Reads number	12000000	12000000	11115006	12000000	12000000	10254848	11160428
Clean data	11375873	11560564	10591620	11305200	11450532	9936370	10610254
	94.80%	96.34%	95.29%	94.21%	95.42%	96.89%	95.07%
average length	100	100	100	100	100	100	100

**Fig 2:**
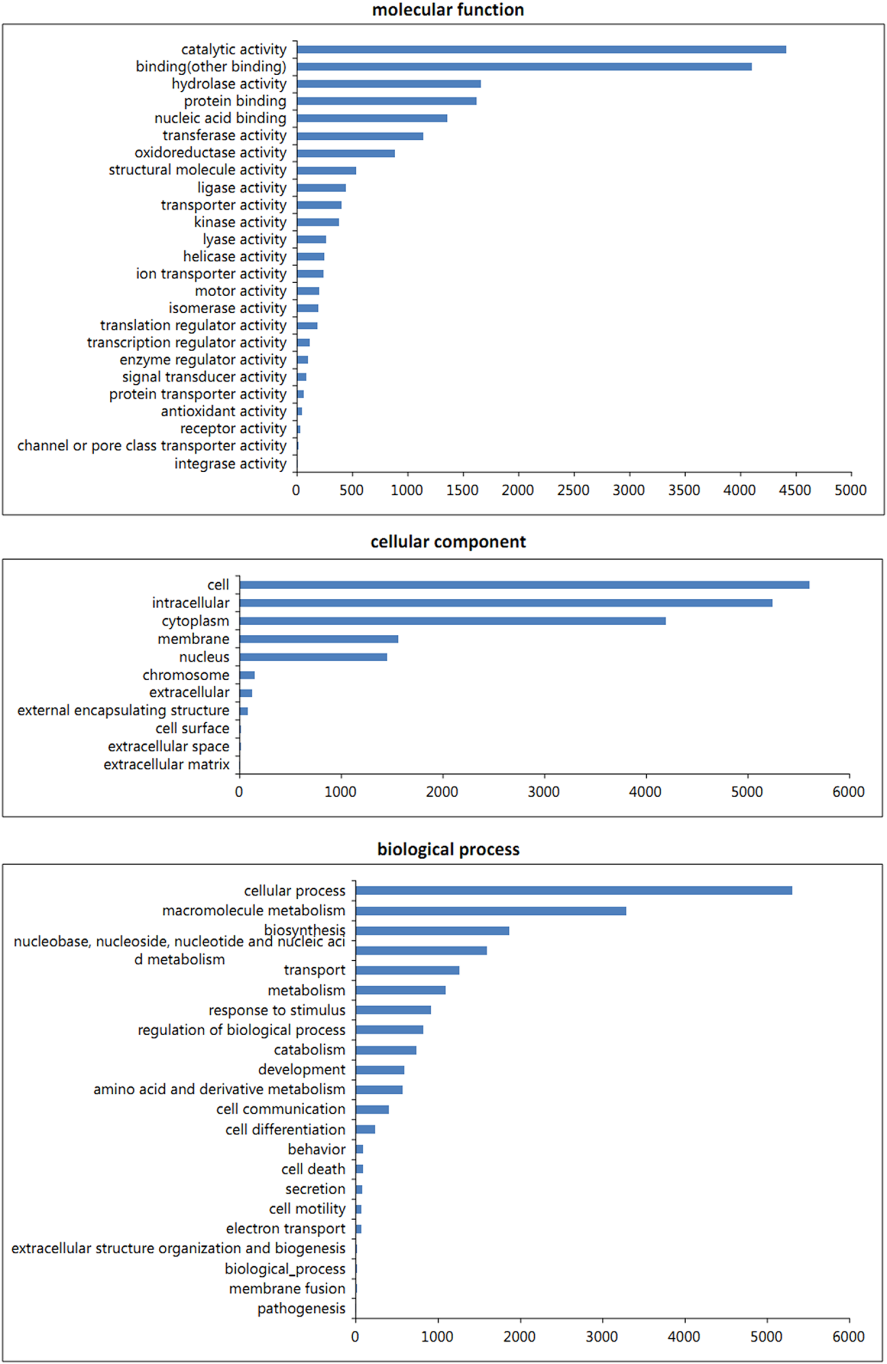
Gene Ontology (GO) classification of unigenes in *H*. *pluvialis*: molecular function (A), cellular component (B) and biological process domains (C). (The -ais indicates the subcategories and the y-ais indicates the number of unigenes).

Based on the gene2go analysis, there were 56,077 unigenes (51.7%) and/or 6,812 protein-encoding genes matching 39,522 Gene Ontology (GO) terms. These unigenes were classified into 3 categories: cellular component, biological process and molecular function, which included 58 subcategories in total (Fig 2). The top five identified subcategories were cell, cellular process, intracellular, catalytic activity and cytoplasm, which possessed 5600 (~9.99%), 5302 (~9.45%), 5242 (~9.35%), 4407 (~7.86%) and 4195 (~7.48%) unigenes, respectively. All the unigenes were further analyzed via KEGG pathway prediction (http://www.genome.jp/kegg/), aimed at interpreting the majoring biochemical pathways and signal transduction involved in astaxanthin inductions. KEGG pathway analysis is an alternative approach for categorising gene functions with an emphasis on biochemical pathways [2]. It showed that upon JA and SA treatments there were a total of 8,668 unigene-related proteins and/or 22 metabolic pathways identified in *H*. *pluvialis* (Figs A and B in S1 File). It was indicated that there were more protein-encoding genes involved in the metabolism of carbohydrate, energy and amino acids once *H*. *pluvialis* cells were exposed to JA and SA inductions. In addition to the signal transduction, the results revealed that the stress response to the hormone induction in *H*. *pluvialis* was also more relevant to cell growth/death of cellular processing and genetic translation information. This is consistent with our previous study on salinity stress where *Haematococcus* cells transformed to aplanospores with up-regulations of carotenogenesis genes, along with astaxanthin accumulation [15].

### The transcription factors involved in hormone stress

Many proteins serve as regulators and transcription factors by gene expression and associated signaling pathways, so the transcription factors (TFs) play very important roles in responding to environmental stress and controlling development [9,12]. In alignment with the regulation of metabolic pathways, theoretically, over-expression of TFs is more precise to indicate overproduction of target bioproducts [16].

The comparative transcriptome analysis in *H*. *pluvialis* between control and hormone induction highlighted 59 unigenes as putative transcription factors in response to hormone stress. They were assigned to 26 families (Table 2). It is worth noting that there are 12 plant-specific TF families: MYB, bHLH, bZIP, C2H2, C3H, E2F-DP, HSF, MADS, Jumonji, SET, WRKY and ERF. Based on the *Arabidopsis* Gene Regulatory Information Server (AGRIS) Database, they possess different biofunctions. For example, zinc finger proteins (incl. C2H2) were responsible to environmental stimuli [17] and regulate diver cell functions like proliferation and apoptosis [12]; basic region/leucine zipper motif (bZIP) could regulate pathogen defense, light and stress signaling in plants [12]. Similarly, the AP2 family was involved in the regulation of disease resistance pathways [18, 19]. The ethylene response factor (ERF) and WRKY played a role in regulating biotic and abiotic stress response [18, 20, 21], including plant hormones challenges of JA, SA and ethylene (ET) [9, 18]. It has been reported that E2F-DP, TBP, Jumonji, and SET TFs were of minor importance in microalgae [16], whereas some (e.g. MYB, AP2, ERF, WRKY, HSF) were associated with hormone signaling in green microalgae *Chlamydomonas reinhardtii* [22].

**Table 2:** Up and down regulated genes involved in carotenoid pathway analysis.

KEGG Orthology category description	JA1-N	JA6-N	JA24-N	SA1-N	SA6-N	SA24-N
ZDS, crtQ; zeta-carotene desaturase [EC:1.3.5.6]	**/**	**up**	**up**	**/**	**/**	**up**
LUT1, CYP97C1; carotene epsilon-monooxygenase [EC:1.14.99.45]	**/**	**/**	**/**	**/**	**/**	**/**
ZEP, ABA1; zeaxanthin epoxidase [EC:1.14.13.90]	**/**	**down**	**/**	**/**	**/**	**down**
Z-ISO; zeta-carotene isomerase [EC:5.2.1.12]	**/**	**/**	**/**	**/**	**/**	**/**
lcyB, crtL1, crtY; lycopene beta-cyclase [EC:5.5.1.19]	**/**	**/**	**/**	**/**	**/**	**/**
LUT5, CYP97A3; cytochrome P450, family 97, subfamily A	**/**	**/**	**/**	**/**	**/**	**/**
(beta- ring hydroxylase) [EC:1.14.-.-]	**/**	**/**	**/**	**/**	**/**	**/**
lcyE, crtL2; lycopene epsilon-cyclase [EC:5.5.1.18]	**/**	**/**	**/**	**/**	**/**	**/**
LUT1, CYP97C1; carotene epsilon-monooxygenase [EC:1.14.99.45]	**/**	**/**	**/**	**/**	**/**	**/**
PDS, crtP; 15-cis-phytoene desaturase [EC:1.3.5.5]	**/**	**/**	**up**	**/**	**/**	**up**
crtZ; beta-carotene 3-hydroxylase [EC:1.14.13.129]	**/**	**up**	**up**	**/**	**/**	**up**
crtISO, crtH; prolycopene isomerase [EC:5.2.1.13]	**/**	**/**	**/**	**/**	**/**	**/**
crtB; phytoene synthase [EC:2.5.1.32]	**/**	**up**	**up**	**/**	**/**	**/**

Apparently, these 26 TFs have a high possibility to participate in SA- and JA-dependent signal pathways in *H*. *pluvialis*. Moreover, they may have different contributions to astaxanthin accumulation in this green microalga, based on their different numbers of unique transcripts (Table 3). The most abundant family was Myb proteins in this study. In higher plants, this family is one of the largest TF families in response to biotic stresses [23], and also a high-level TF can affect other TFs, regulating a broad range of genes [24]. Therefore, the remarkable expression of Myb family in *H*. *pluvialis* is likely to play an important role in enhancing the astaxanthin/carotene biosynthesis. However, further study for the characterisation of these TFs modulating hormone signal pathways is needed on the astaxanthin biosynthesis in *H*. *pluvialis*.

**Table 3:** Transcription factor families identified in *H*. *pluvialis*.

Transcription factor family	No. of unique transcripts	Transcription factor family	No. of unique transcripts
Myb	9	Zinc finger	2
CCAAT	7	MADS	2
GATA	4	MBF1	2
TBP	4	MYB-related	1
E2F/DP	3	NF-YB	1
C3H	2	NF-YC	1
NF-X1	2	TALE	1
ERF	2	HSF	1
Jumonji	2	B3	1
M-type	2	SNF2	1
SET	2	WRKY	1
bZIP	2	bHLH	1
AP2	2	C2H2	1

### Differential expressed genes of *H*. *pluvialis* upon JA and SA inductions

**Fig 3:**
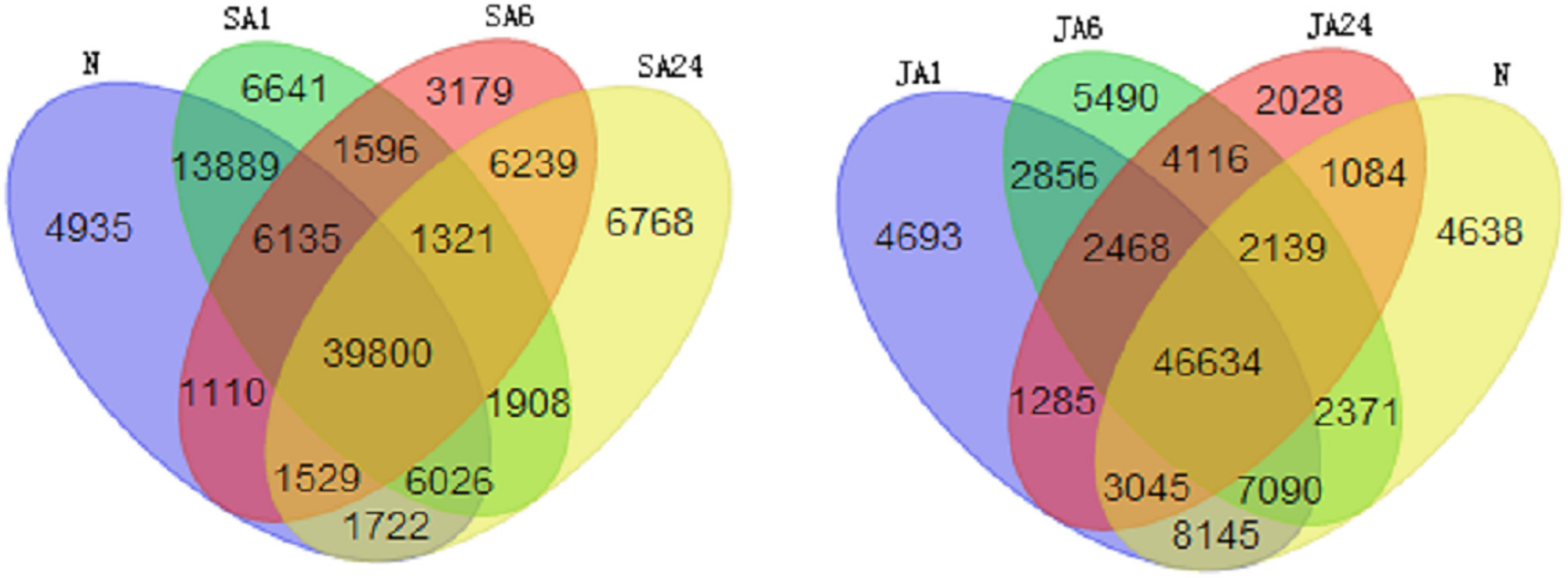
Venn diagrams showing genes unique and shared between different treatments in *Haemotococcus pluvialis*. Each colour represents a sample (as labelled) and the numbers indicate the amount of genes in the region. (A) Comparative result of SA-treated samples (SA1, SA6 and SA24) and the control (N); (B) Comparative result of JA-treated samples (JA1, JA6 and JA24) and the control (N).

When the abundances of transcripts were normalised into RPKM, it showed that the different-expressed genes in control, SA and JA-induced groups, were 75,146, 102,798 and 98,082 unigenes, respectively. In both JA and SA treatments, the mount of differential expressed genes also varied between different sampling times (Fig 3). There were 77316, 60909 and 65313 unigenes identified in SA1, SA6 and SA24, respectively but each individual had 65850 (87.6%), 48574 (64.6%) and 49077 (65.3%) genes shared with the control. Under the JA induction, there were were 76216, 73164, 62799 unigenes in JA1, JA6 and JA24, respectively and they had 64914 (86.4%), 58234 (77.5%) and 52902 (70.3%) unigenes similar to the control (Fig 3). These results demonstrate that longer treatment times could induce more differential expressed genes in both JA and SA treatments. Moreover, the number of differential unigenes continuously increased under the JA treatment, but became stable or slightly declined after 6 hrs in the SA treatment. However, they both showed that the most dynamic change happened after 6 hrs.

**Fig 4:**
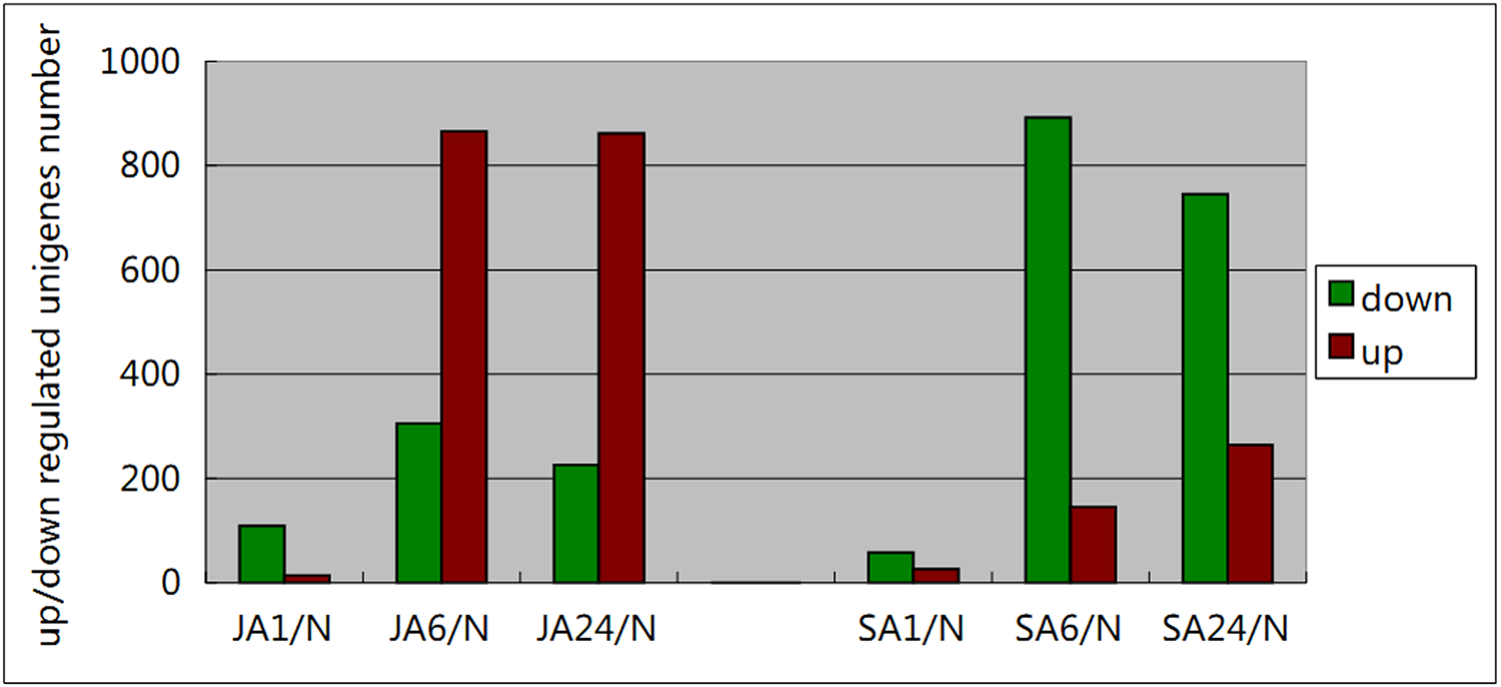
The number of up-regulated (red) and down-regulated (green) unigenes in *Haematococcus pluvialis* on JA and SA treatments.

The significant differential-expressed genes were further identified with the standard of both q value (FDR, false discovery rate) <0.001 and p value <0.001. Based on these significant values, the number of differential up-regulated genes gradually increased from SA1 to SA24, showing the maximum number of 264 unigenes in SA24 (Fig 4). However, the most up-regulated genes in JA samples occurred in JA6 with 889 unigenes, increasing sharply from 14 of JA1 then reducing slightly to 862 of JA24. A similar pattern was also observed on those down-regulated genes in JA-induced groups. It also peaked in JA6 (305). The down-regulated genes in SA-induced groups varied differently from up-regulated genes. They dramatically elevated from SA 1 (58), reaching the maximum level in SA6 (892), then declined in SA24 (745). These results further revealed that the JA and SA treatments had induced different genetic regulations on *H*. *pluvialis*. In comparison, JA induction had more up-regulated unigenes, whereas SA treatment had more down-regulated unigenes.

**Fig 5:**
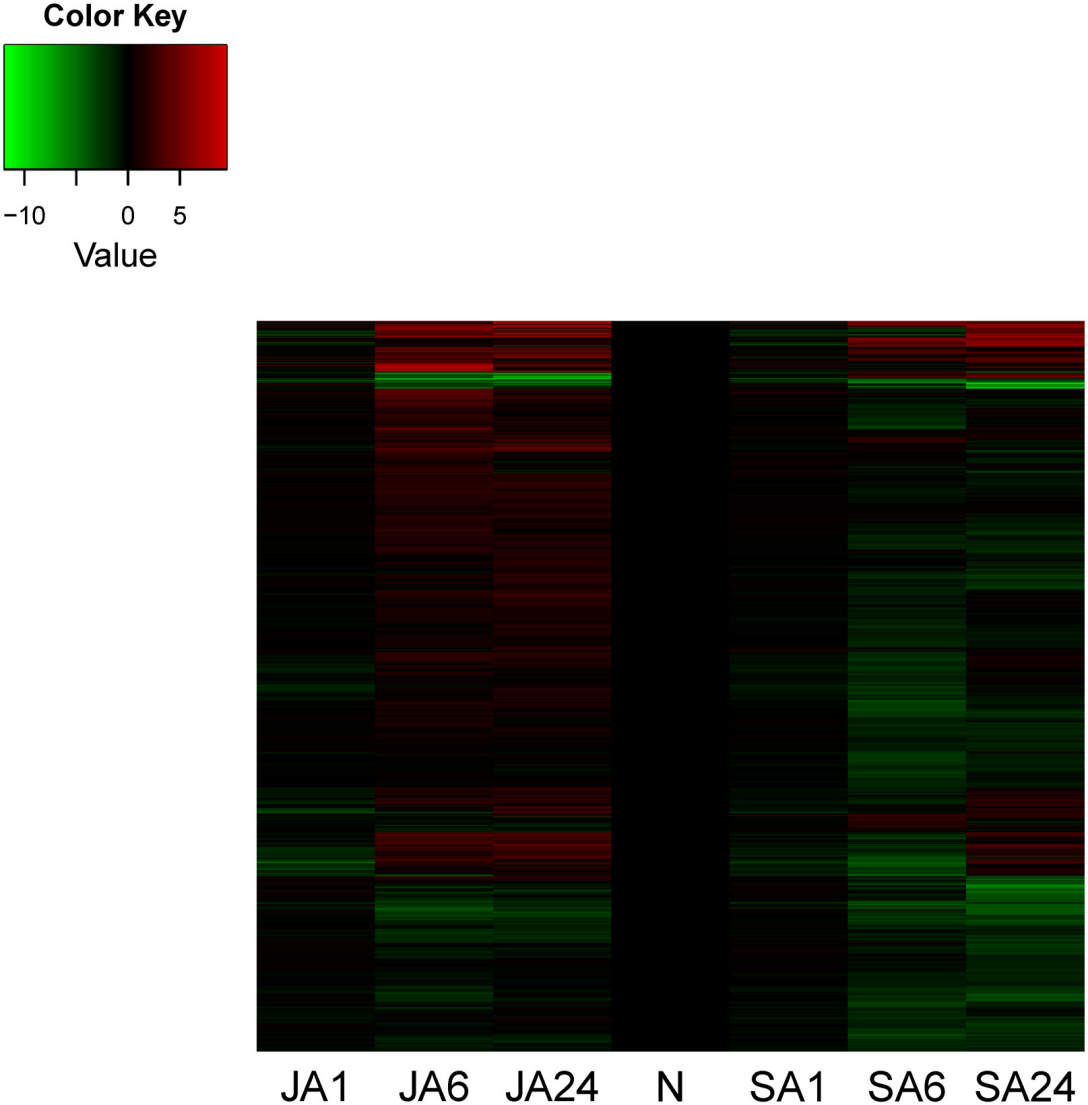
Heatmap of differentially epressed genes in *Haematococcus pluvialis* under SA and JA treatments. Each line represents an individual gene; the red and green lines represent the relative rise level and decline level of gene epression compared to the control level.

This result was consistent with the hierarchical cluster analysis using heatmap, showing the gene expressed profiles were most different (up-and down-regulations) at SA6 and JA6 (Fig 5). Similarly, another study on the red seaweed *Chondrus crispus*, in response to Methyl Jasmonate (MeJA) treatment, also showed most dynamic responses occurred after 6 h [25]. Such a delay is probably related to these chemicals needing a bit of time to penetrate the algal cell walls. The analysis on the differential expressed genes revealed that hormone-induce-related unigenes in *H*. *pluvialis* were more up-regulated in the JA treatment and down-regulated in SA treatment, and they become abundant between 6 and 24 hrs.

### Carotenoid metabolic difference between JA and SA inductions

Through the analysis of a pairwise samples comparison, there were 12 unigenes identified in the carotenoid metabolism (Table 3). As stated in previous literature, the reaction from two GGPP molecules into phytoene was catalysed by phytoene synthase (*crtB/psy*) [15]. The successive desaturation reactions were phytoene catalysed by phytoene desaturase (*pds*) and ζ-carotene desaturase (*zds*), then the cyclization of lycopene into β-carotene was catalysed by lycopene β-cyclase with *crt*L-b (*lcy*) gene encoding. The *crt*R-b was responsible for adding two hydroxy groups to C3 and C3' of β-carotene [26]. Zeaxanthin epoxidase (*zep*) was responsible for the epoxidation of zeaxanthin, catalysing the conversion of zeaxanthin to antheraxanthin and violaxanthin [27]. The carotene isomerase (Z-ISO) could catalyse *cis* to *trans* conversion of carotenes [28]. One of carotene isomerases (*crt*ISO) also had a similarity to *cis*-to-*trans* isomerisation [29]. The pro-lycopene was further isomerized by *crt*ISO to yield all-*trans*-lycopene, and then cyclized to be β-carotene [30]. In this study, only 5 genes (*zds*, *pds*, *crt*Z, *crt*B and *zep*) showed significantly differential expressions (Table 2), and also some differences were detected on the expression patterns of these unigenes between JA and SA treatments.

In comparison, these unigenes were regulated earlier in JA treatment. Their differential expressions started in JA6 and were sustained to JA 24, whereas it was only detected in SA24. The gene *crt*B was up-regulated in the JA treatment, rather than in the SA treatment. Zeaxanthin epoxidase (ZEP) was down-regulated in both treatments, but at different times (JA6 vs. SA 24). In this regard, it seems that the carotenoid metabolism was modulated earlier by JA induction, probably more intensive as well.

### Analysis of the transcriptome involved in astaxanthin biosynthesis

**Fig 6:**
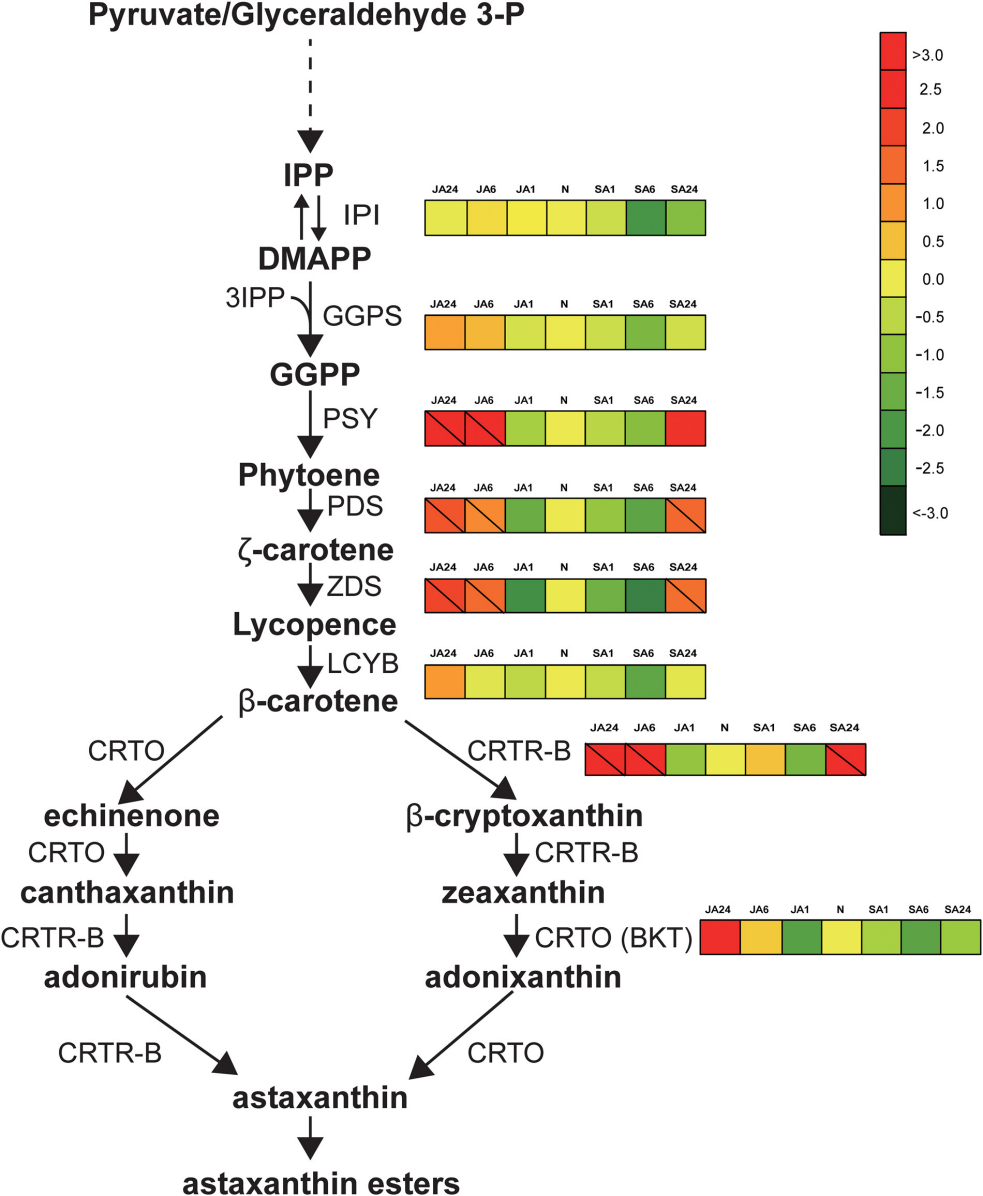
Differential epression of genes involved in the carotenoid biosynthesis pathway of*Haematococcus pluvialis*. The color chart represents up- (red) and down-regulated (green) folds. The slash in the square indicates a significant differential-epression at the level of > 2 folds, FDR < 0.001, RPKM > 20. IPI- isopentenyl diphosphate isomerase; GGPS- geranylgeranyl diphosphate synthase; PSY- phytoene synthase; PDS- phytoene desaturase; ZDS- zeta-carotene desaturase; LCYB- lycopene beta-cyclase; CRTR-B- carotenoid hydroylase; BKT/CRTO- beta-carotene ketolase.

The astaxanthin biosynthetic pathway is presented in Fig 6. DMAPP synthesis is thought to be catalysed by isopentenyl diphosphate isomerase (IPPI) [31]. However, two putative IPPI transcripts were not significantly up-regulated in the first 24h after JA or SA addition, which was also consistent with our previous studies [6, 7]. In the SA treatment, the transcripts of most carotenogenetic genes, including the important astaxanthin synthetic genes of *ggps*, *lcyb* and *crtO(bkt)*, did not change or down-regulated compared to the control level. In the JA treatment, these genetic transcript levels in JA1 were slightly down-regulated compared to the control level. But all the carotenogenetic genes responsible for the astaxanthin biosynthesis from DMAPP were up-regulated in JA6 and JA24 (except for *lcyb*). These results were consistent with our previous results of RT—qPCR [6, 7] that genetic up-regulation (incl. *pds*, *crt*R-B and *bkt*/*crt*O) in astaxanthin biosynthesis occurred at 6 and 24 hrs upon JA and SA hormone inductions, respectively.

The transcriptome analysis showed that 4 up-regulated genes (*psy*, *pds*, *zds* and *crt*R-B) exhibited notably different levels in JA24 (Fig 6). In contrast, only 3 genes (*pds*, *zds*, *crt*R-B) were up-regulated at significantly different levels in SA24. For example, the transcripts of *psy* gene was >3-fold more abundant in JA6, JA 24 and SA24 than the control levels. In addition, two genes, *pds* and *zds*, were also up-regulated by 1.0- to 2.0-fold in samples of JA6, JA 24 and SA24. The RNA-seq analysis also showed that two genes of *bkt*/*crt*O, encoding the rate-limiting steps converting β-carotene to astaxanthin, were up-regulated (>3.0-fold) in JA24, whereas the transcripts of *crt*R-B increased >3-fold in JA6, JA 24 and SA24. Our previous studies have confirmed that SA and JA inductions resulted in a higher astaxanthin production at 1.63 and 1.458 mg/L, respectively [6, 7]. So these genes were responsible for supplying different units for astaxanthin synthesis under hormone stress. In other previous reports, these genes were also up-regulated at the mRNA level when *H*. *pluvialis* were exposed to high light and salinity for astaxanthin stimulation [15, 32–34]. Therefore, these genes can play a prominent regulatory role in astaxanthin biosynthesis, and the up-regulation of these carotenogenic genes can result in astaxanthin rapid accumulation.

## Materials and Methods

### Cell culture and SA (or JA) treatment

Strain 712 of *H*. *pluvialis* was cultured in 500mL beakers, in MCM medium without aeration, under a light intensity of 25 mmol photons m^−2^s^−1^ with a 12h: 12h light/dark cycle at 22°C [6, 7]. When the cells reached the logarithmic phase (10^5^ cells ml^-1^), the culture was evenly divided into seven aliquots of 1000mL each. They were used as an experimental control (N, without addition of JA or SA), three salicylic acid (SA) treatments and three jasmonic acid (JA) treatments. The SA and JA dosages were at 25 mg/L for the induction [6, 7], and the hormone-induced cultures were sampled at 1, 6 and 24 hrs, respectively. All the samples (JA1, JA6, JA24, SA1, SA6, SA24 and control N) were centrifuge-harvested at x 8000 g, kept at 4°C for 10 min, and then stored at −80°C till subsequent analyses.

### RNA extraction

The frozen *H*. *pluvialis* cells were ground using a mortar and pestle on a tope of dry ice. Total RNA of algal cells was extracted using Trizol reagent [6, 7], digested by 10 u DNaseI (TaKaRa, Japan) at 37°C for 30min, then purified via the Dynabeads^®^ Oligo (dT) 25 kit (Life, America) according to the manual. In each sample, 100 ng of mRNA was used as a template for cDNA library construction, as below:

### cDNA library construction

cDNA synsthesis was conducted using NEBNext^®^ UltraTM RNA Library Prep Kit (NEB, America) according to the method used by Ng et al. [35]. Random Primers and first Strand Synthesis Reaction Buffer (5X) were used to crack mRNA at 94°C for 15 min. The first-strand cDNA was produced by adding Murine RNase Inhibitor and ProtoScript II Reverse Transcriptase, and subsequently incubated at 25°C for 10 min, 42°C for 50 min and 70°C for 15min. The double-stranded cDNA was synthesised by incubating with Second Strand Synthesis Reaction Buffer (10×) and Second Strand Synthesis Enzyme Mi at 16°C for 1h. The double-stranded cDNA ends were blunted at 20°C for 30 min, and then at 65°C for another 30 min. The short adaptors were ligated on both ends at 20°C for 15 min. Purification with size selection (200~500bp) was conducted using AMPure P Beads (Beckman Coulter, Brea, CA, USA). Finally, the purified samples were amplified via PCR reactions: 98°C, 10 Sec; 12–15 cycles of 98°C for 10 sec, 65°C for 30 sec and 72°C for 30 sec. After Onedrop quantification, the sequencing library was further purified by 2% agarose gel electrophoresis detection and high-sensitivity DNA chip detection.

### Illumina sequencing, assembling and annotation

Paired-end sequencing of cDNA was carried out with the Illumina Hiseq2000 platform by Shanghai Hanyu Bio-Tech. Based on quality, the clean reads were filtered from raw data then assembled into contiguous sequences (contigs) by Trinity software (version 2013-02-25). These contigs were used for gene prediction by GetORF (EMBOSS http://emboss.open-bio.org/) [36]. Similar protein-encoding genes were selected and subsequently blasted with the references of protein-encoding sequence from the NR of GenBank, Gene Ontology (GO), Kyoto Encyclopedia of Genes and Genomes (KEGG), and Kyoto Orthology Genome (KOG) identifiers using Blastp. The matching annotated coding sequences were analysed for GO classification (http://www.geneontology.org/), pathway construction, epression abundance and difference analysis using GoPipe (E-value < 10^−5^) [37]. The abundances of transcripts were normalised as reads per kilobase of eonmodel per million mapped reads (RPKM). The associated metabolic pathways were analyzed and predicted by KEGG mapping [38, 39]. Based on the KOG data, putative transcription factors (TFs) were identified by searching the Arabidopsis Gene Regulatory Information Server (AGRIS) Database [12].

### Data analysis

In order to identify key regulatory genes and associated metabolic pathways involved in the hormone stress response of *H*. *pluvialis*, the comparative transcriptome analysis was carried out between control and hormone treatment groups. Meanwhile, the comparison of SA and JA samples also highlighted the differential genetic epressions in carotenoid and astaanthin biosynthesis metabolism. The comparative analysis was conducted by Student T tests on Ecel. A significance level of 0.001 was used for all tests.

## Conclusions

Summarily, time course-dependent changes in the transcriptome of unsequenced green microalga *H*. *pluvialis* showed not only a transcriptomic RNA-seq dataset, but also biological insights on the epression patterns of contigs associated with astaanthin metabolism under JA and SA inductions. A large number of related enzymes and transcription factors coding genes were identified in the carotenoid and astaanthin metabolisms. Based on 12 unigenes involved in the carotenoid metabolism, the epression patterns of 5 genes (*zds*, *pds*, *crt*Z, *crt*B, *zep*) were different between JA and SA inductions. The up-regulation of carotenogenic genes of *psy*, *pds*, *zds* and *crt*R-B correlated to ecessive astaanthin accumulation in *H*. *pluvialis* but they showed different up-regulated patterns with stress of the same level of JA or SA inductions, suggesting different metabolic pathways were involved and also different amounts of astanathin accumulation. As a pioneer study, the derived transcriptomic data and the contigs can facilitate comparative transcriptomic studies on *H*. *pluvialis* with other different stresses. Although it is the first time that some plant-specific transcription factor families (e.g., MYB, AP2/ERF, WRKY, HSF and etc) were identified in *H*. *pluvialis* in response to hormonal (JA and SA) stress, their known biofunctions can provide valuable information for directing metabolic engineering efforts towards higher astaanthin accumulation in *H*. *pluvialis*. Therefore, an in-depth functional analysis of these TFs is needed to further investigate their unique roles in stress response of *H*. *pluvialis*, coupled with astaanthin production.

## Supporting Information

S1 File The number of protein-encoding genes involved in different metabolic pathways. The left column shows different metabolic pathways and the numbers and x-axis represents the number of genes in each relevant pathway (**Figure A**). Number of genes up- or down-regulated in different metabolic pathways of *Haematococcus pluvialis* upon phytohormone induction. SA treatment (A and C); JA treatment (B and D). **(Figure B)**.
(DOC)
