# Supplementary material for: Transcriptome Analysis in Haematococcus pluvialis: Astaxanthin Induction by Salicylic Acid (SA) and Jasmonic Acid (JA)
Source: PLoS One. 2015 Oct 20;10(10):e0140609. doi: 10.1371/journal.pone.0140609 (PMC4979887; doi:10.1371/journal.pone.0140609)
Supplement: S1 File — The left column shows different metabolic pathways and the numbers and x-axis represents the number of genes in each relevant pathway (Figure A). Number of genes up- or down-regulated in different metabolic pathways of Haematococcus pluvialis upon phytohormone induction. SA treatment (A and C); JA treatment (B and D). (Figure B). (DOC) [file pone.0140609.s001.doc]

**Supporting Information 1**

**Supporting Information Captions**

**S1 File.** The number of protein-encoding genes involved in different metabolic pathways. The left column shows different metabolic pathways and the numbers and x-axis represents the number of genes in each relevant pathway (**Figure A**). **Number of genes up- or down-regulated in different metabolic pathways of *Haematococcus pluvialis*** upon phytohormone induction. SA treatment **(**A and C); JA treatment (B and D) **(Figure B)**.


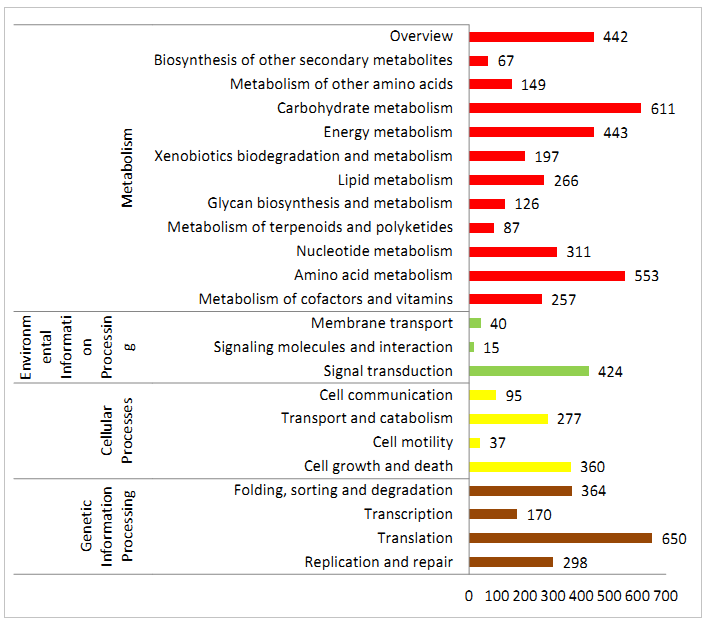


**Figure A**

**
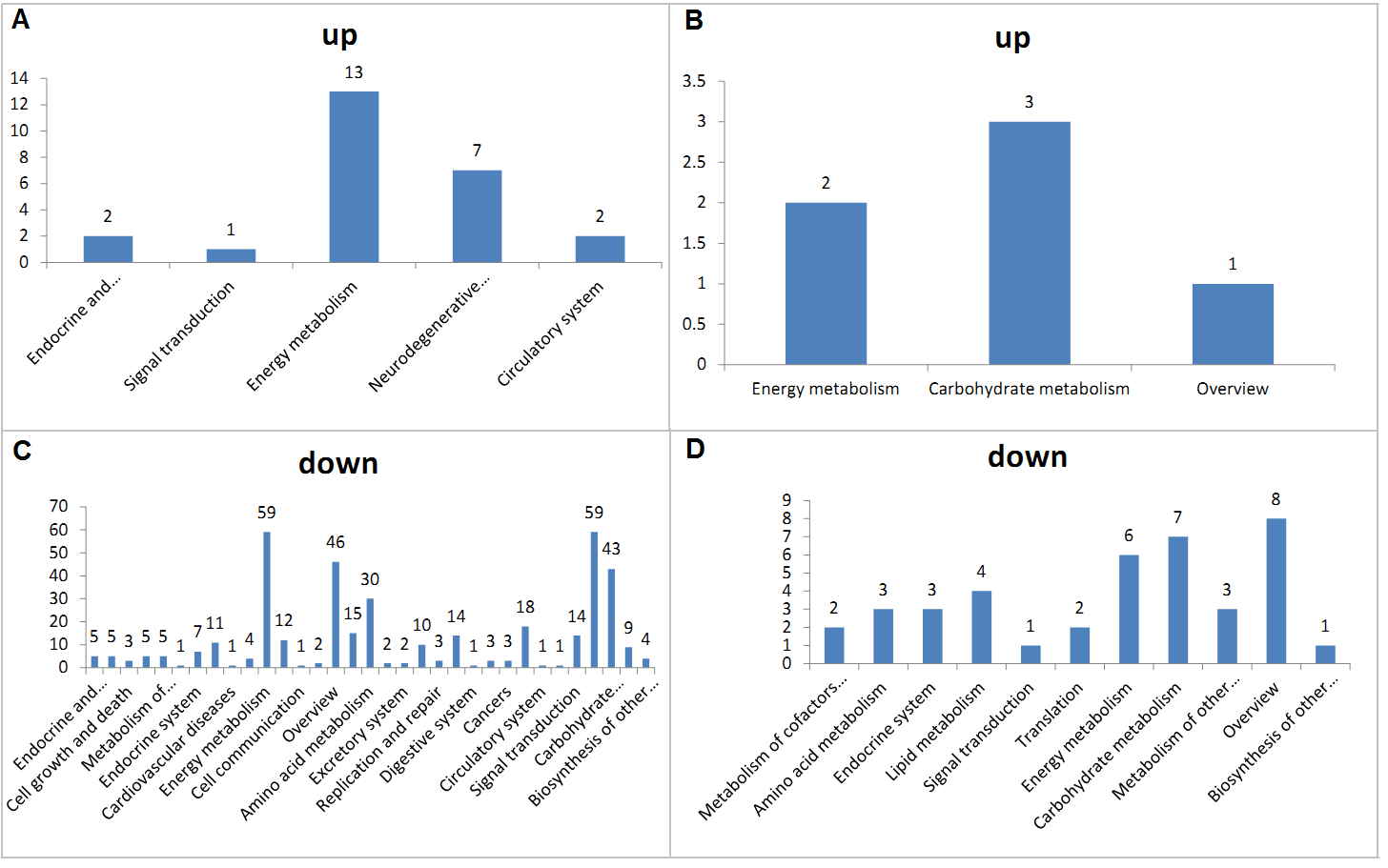
**

**Figure B**
